# Soybean-*Bupleurum* Rotation System Can Optimize Rhizosphere Soil Microbial Community via Impacting Soil Properties and Enzyme Activities During *Bupleurum* Seedling Stage

**DOI:** 10.3390/microorganisms13102346

**Published:** 2025-10-13

**Authors:** Qingshan Yang, Peng Dong, Mengni Chen, Hui Wang, Lu Wang, Jiawei Yuan, Chengyu Hu, Zhen Liu, Yongshan Li, Qiaolan Fan

**Affiliations:** 1 Cotton Research Institute, Shanxi Agricultural University, Yuncheng 044000, China; 2 College of Agriculture, Shanxi Agricultural University, Taiyuan 030031, China; 3 College of Resources and Environment, Henan Agricultural University, Zhengzhou 450002, China

**Keywords:** preceding crop, *Bupleurum*, soil property, enzyme activity, soil microorganisms

## Abstract

To avoid continuous cropping problems with *Bupleurum*, we screened suitable preceding crops for rotation with *Bupleurum* through different crop rotations. Therefore, the objective of this study was to find out the relationships between microbial community characteristics, soil properties, and enzyme activities under four different rotation patterns, including fallow-*Bupleurum* (CK), maize-*Bupleurum* (M), soybean-*Bupleurum* (So), and sunflower-*Bupleurum* (Su). Results indicated that under all four rotation patterns, So treatment significantly enhanced soil nutrients and enzyme activities compared to CK. So not only optimized the composition of soil bacterial and fungal communities but markedly enhanced microbial α diversity. Additionally, So exhibited high similarity in bacterial and fungal community composition with M, and featured complex symbiotic relationships within the soil microbial network. While no clear discrepancies were detected in the abundance of the top twenty metabolic pathways in the predictive functions of bacterial and fungal communities across four rotation patterns, the metabolic pathway function MET-SAM-PWY (methionine synthesis pathway) in bacterial communities and the metabolic pathway function VALSYN-PWY (valine synthesis pathway) in fungal communities were particularly prominent under the So rotation pattern. RDA suggested that soil properties (available phosphorus and pH) and enzyme activities (sucrase and alkaline phosphatase activities) were the driving forces for bacterial community composition, while soil properties (soil organic matter and available potassium) and enzyme activities (sucrase and catalase activities) regulated fungal community composition. Hence, the soybean-*Bupleurum* rotation pattern represents a cultivation practice more beneficial for the sustainable development of the bupleurum industry, which can significantly improve soil fertility and the micro-ecological environment.

## 1. Introduction

*Bupleurum* is considered to be one of China’s traditional bulk medicinal herbs. As a perennial medicinal plant, the dried roots of *Bupleurum* are used to disperse wind, resolve exterior syndromes, soothe the liver, relieve depression, and elevate yang energy [1]. Currently, *Bupleurum* faces enormous market demand, yet wild resources are increasingly scarce, making artificial cultivation its primary source. Given its designation as a production area for *Bupleurum*, Shanxi Province is confronted with significant challenges related to continuous cropping, namely the expansion of cultivation scale and the scarcity of arable land resources. These issues are evident in the worsening of root rot, the decline in plant vigor, the frequent death of seedlings, and the root decay, which severely impacts the yield and quality of *Bupleurum* [2,3]. This hinders the sustainable development of local *Bupleurum* cultivation. Therefore, implementing a rational crop rotation system is crucial for alleviating these obstacles and promoting the healthy development of the *Bupleurum* industry.

Previous studies have demonstrated that long-term monocropping can cause changes in the physicochemical properties and enzyme activity of soil, resulting in imbalances in soil microecology [4]. Consequently, it impacted soil health and sustainability [5]. An ecologically efficient cultivation model was represented by rational crop rotation, particularly when combining fibrous rooted crops with taproot medicinal plants, which improved soil physicochemical properties [6]. Zhao et al. [7] demonstrated that compared to continuous cropping, crop rotation impacted soil hydrolytic enzyme activity by altering nutrient levels in the soil. Li et al. [8] found that rotating crops of astragalus and oats markedly rose the content of soil organic matter and enzyme activity. The activities of sucrase, urease, acid phosphatase, and catalase in the soil were found to increase markedly by Qin et al. [9] as a result of pineapple–sugarcane rotation. Soil enzyme activity was enhanced, which sped up the breakdown of organic material and the movement of nutrients around the soil, thus enhancing soil fertility and quality [10]. Thus, it is particularly crucial to select suitable preceding crops in a rotation system [11]. The root residues and secretions from different crops not only improved soil structure, regulated the microenvironment, optimized nutrient distribution, and provided favorable conditions for soil microbial communities, but promoted the growth of subsequent crops [12].

Soil microorganisms performed a pivotal function in the cycling of soil nutrients, the decomposition of organic matter, and the enhancement of crop resilience to stress [13]. However, continuous cropping over multiple years lead to decrease soil enzyme activity, as well as a reduction in beneficial microbial abundance and an increase in pathogen populations. The original plant–microbe interaction network was disrupted by this, with normal plant growth and development ultimately being inhibited [14]. The type of crop and the systems of cultivation had a considerable effect on the soil microbial community composition and their interactions [15]. Appropriate crop rotation can significantly reduce the amount of soil pathogens, improve the structure and diversity of soil microorganisms [16], and enhance the complexity of microbial networks [17]. Studies by Zhang et al. [18] (maize–tobacco rotation) and Liu et al. [19] (fennel–notoginseng rotation) indicated that crop rotation enhanced soil bacterial abundance and diversity, while suppressing the proliferation of harmful fungi. Nevertheless, Wang et al. [20] discovered through a thirty-year rotation trial that crop rotation did not significantly impact soil bacterial diversity. Most studies currently focus on comparing the structures of microbial communities under different crop rotation systems versus continuous monoculture. So far, few studies have explored how different preceding crops affected soil microbial community characteristics in *Bupleurum* fields.

Therefore, in this study, high-throughput sequencing technology combined with random forest algorithms and network topology analysis were employed to systematically compare the effects of three preceding crops on rhizosphere soil properties, enzyme activities, and microbial community structure during the seedling stage of *Bupleurum*. It further explored the relationships among these factors to identify suitable preceding crops, optimized the crop–bupleurum rotation system, and provide theoretical support for the sustainable cultivation of *Bupleurum*. Herein, we proposed the following hypotheses: (1) The soybean-*Bupleurum* rotation pattern can improve soil physicochemical properties and enhance soil enzyme activities; (2) The soybean-*Bupleurum* rotation pattern helps optimize soil microbial community structure and increase functional prediction abundance.

## 2. Materials and Methods

### 2.1. Site Description

The research was conducted from June to October 2023 at the experimental fields of the Cotton Research Institute Farm, Shanxi Agricultural University (35°02′ N, 110°58′ E), at an elevation of 376 m. The region exhibits a continental temperate monsoon climate with an annual average precipitation of 564 mm, concentrated from July every year to September. The annual average temperature is 13.3 °C, with a frost-free period of around 190 days. The soil at the test site is loam. Additionally, soil physicochemical properties at a depth of 0–20 cm were as follows: organic matter content 2.07 g·kg^−1^, alkali-hydrolysable nitrogen content 33.95 mg·kg^−1^, available phosphorus content 49.73 mg·kg^−1^, available potassium content 113.24 mg·kg^−1^, pH 8.98.

### 2.2. Experimental Design

The experiment was designed using a single-factor completely randomized design. Four different crop rotation patterns were established: fallow-*Bupleurum* (CK), maize-*Bupleurum* (M), soybean-*Bupleurum* (So), and sunflower-*Bupleurum* (Su). Each treatment was replicated three times, with plots measuring 2.5 m × 15 m. The test materials comprised maize, soybean, and sunflower varieties were ‘Zhengdan 958’, ‘Yundou 101’, and ‘Oil sunflower dwarf large head H667’, respectively. The preceding crops were sown in June 2023 using the hole-sowing method. Field management followed the growth habits of each crop and crop residues were removed from the field after harvest. *Bupleurum* was sown in August 2023 using the row-seeding method between the crop rows.

### 2.3. Soil Samples Collection and Analysis

In October 2023, soil samples were collected from the 0–20 cm soil layer within a 0–5 cm radius around *Bupleurum* roots during the seedling stage. The ‘S’-shaped sampling method was used. We removed debris such as stones and plant residues. One portion of the samples was placed in ice boxes and transported to the lab for storage at −80 °C in order to determine soil microbial community characteristics. Another portion was air-dried to determine soil properties and enzyme activities.

Soil organic matter (SOM) was determined using the potassium dichromate oxidation titration method [21]; ammonium nitrogen (NH_4_^+^-N) was measured via continuous flow analysis [22]; available phosphorus (AP) was assessed using the sodium bicarbonate extraction-molybdenum antimony colorimetric method [23]; available potassium (AK) was measured via ammonium acetate extraction followed by flame photometry [24]. Soil pH was tested using a pH meter (water:soil = 2.5:1). Soil catalase activity (CAT) was assessed through potassium permanganate titration [25]; urease (UR) was analyzed via the phenol-sodium hypochlorite colorimetric method [5]; sucrase (SC) was determined using the 3,5-dinitrosalicylic acid colorimetric method [25]; alkaline phosphatase (ALP) was analyzed using the sodium phosphate colorimetric method [26].

### 2.4. High-Throughput Sequencing and Sequence Analysis

The CTAB method was employed to obtain genomic DNA from the soil samples, after which PCR amplification was carried out. Following electrophoretic detection, magnetic bead recovery, concentration measurement, and pooled sample purification, sequencing was performed using a MiSeq high-throughput sequencer (Illumina, San Diego, CA, USA). The V3–V4 region of the bacterial 16S rRNA gene (primers 341F: 5′-CCTAYGGGRBGCASCAG-3 and 806R: 5′-GGACTACNNGGGTATCTAAT-3′) and the ITS1–5F region of the fungal ITS rRNA gene (primers ITS5F: 5′-GGAAGTAAAAGTCGTAACAAGG-3′ and ITS2R: 5′-GCTGCGTTCTTCATCGATGC-3′) were specifically targeted. The PCR reaction system consists of 15 µL of 2× reaction buffer, 1 µM/µL of forward and reverse primers, 10 µL of gDNA (10 ng), and 2 µL of H_2_O. Reaction protocol: pre-denaturation at 98 °C for 1 min; 30 cycles of 98 °C for 10 sec, 50 °C for 30 sec, and 72 °C for 30 sec; and 72 °C for 5 min.

After performing quality control, trimming, denoising, assembly, and chimera removal on the raw sequence FASTQ files using QIIME2 data2, the final amplicon sequence variants (ASVs) were obtained [27]. Alpha diversity indices (Chao 1 and Shannon indices) were reckoned via the QIIME2 software (v 2022). Shared/unique ASVs between groups were visualized using the ‘VennDiagram’ package in R software (v4.0.3). Beta diversity was assessed based on the Bray−Curtis, and non-metric multidimensional scaling (NMDS) was used to visualize differences in microbial community structure among samples. Functional annotation of the bacterial and fungal community data was performed using the MetaCyc database [28]. Significant differences in metabolic pathways were identified using ANOVA and Duncan’s multiple range test (*p* < 0.05), comparing functional variations in microbial communities between groups. Data were normalized for ANOVA analysis, where positive values represented relative abundance above the mean, and negative values represented relative abundance below the mean.

### 2.5. Statistical Analysis

Using IBM SPSS Statistics (v.27.0) software (IBM Corp, Armonk, NY, USA) to analyze the differences of the soil properties, enzyme activities, and soil alpha diversity under different crop rotations via Duncan’s test (*p* < 0.05). Redundancy analysis (RDA), microbial community composition figure, and network heatmap were analyzed and obtained through the Paisenno Gene Cloud Platform (https://www.genescloud.cn accessed on 6 September 2025) to reveal relationships among soil properties, enzyme activity, and microbial communities. The importance of microbial communities was produced by MicroBio Alliance Life Science Cloud Platform (https://www.bioincloud.tech accessed on 6 September 2025). Soil microbial network topology diagrams were constructed using Gephi (v 0.10.1) software. All other figures in this paper were drawn using Origin (v.2021) and Adobe Illustrator 2021 software.

## 3. Results

### 3.1. Soil Properties and Enzyme Activities

**Figure 1:**
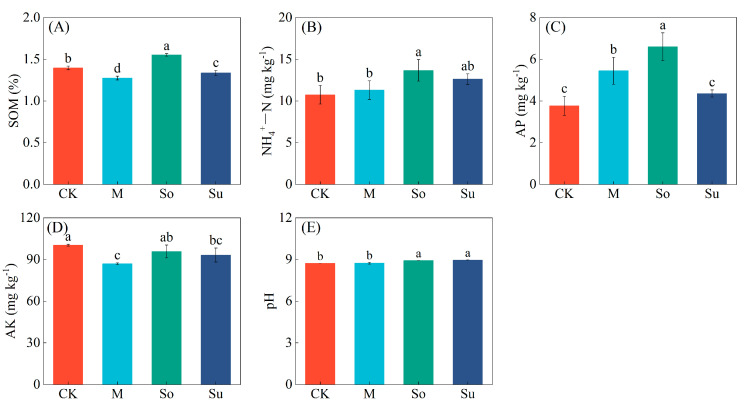
Impact of crop rotation patterns on soil properties in the rhizosphere of *Bupleurum* seedlings. (**A**) soil organic matter (SOM), (**B**) ammonium nitrogen (NH_4_^+^-N), (**C**) available phosphorus (AP), (**D**) available potassium (AK), (**E**) pH. Data represent the mean (*n* = 3) ± standard deviation (SD). Different lowercase letters are significantly different among treatments (*p* < 0.05; Duncan’s test).

Different crop rotation patterns had a significant influence on the properties of the rhizosphere soil during the seedling stage of *Bupleurum* (Figure 1). The content of SOM, NH_4_^+^-N, AP, and pH value was found to be significantly higher in So than in CK, with increases of 11.24%, 27.22%, 75.22%, and 2.18% respectively. Compared with CK, M and Su significantly reduced the content of SOM and AK by 4.33−8.63% and 7.15−13.42%, respectively. The content of AP was significantly higher than CK (44.78%) under M. In contrast with M, Su considerably elevated SOM and pH but considerably decreased AP content.

**Figure 2:**
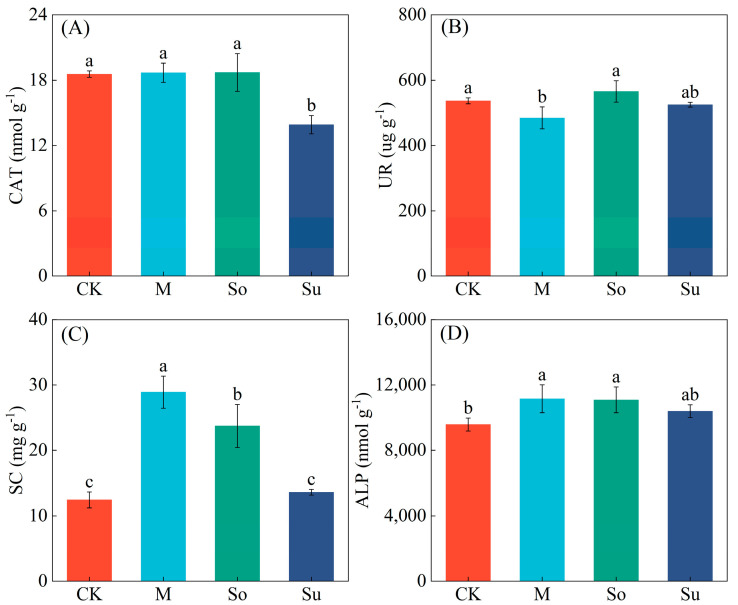
Impacts of crop rotation systems on rhizosphere soil enzyme activity during the seedling stage of *Bupleurum*. (**A**) catalase (CAT), (**B**) urease (UR), (**C**) sucrase (SC), (**D**) alkaline phosphatase (ALP). Data show the mean ± SD. Significant differences among the treatments were indicated by the use of different lowercase letters (*p* < 0.05; Duncan’s test).

As for CAT, compared with Su, CK, M, and So showed significant increases, ranging from 33.42% to 34.49% (Figure 2A). CK and So significantly increased UR by 10.78−16.73% compared to M (Figure 2B). Compared with CK, M and So significantly increased SC (15.78−16.45%) and ALP (90.53−132.18%) (Figure 2C,D).

### 3.2. Soil Microbial Community

**Figure 3:**
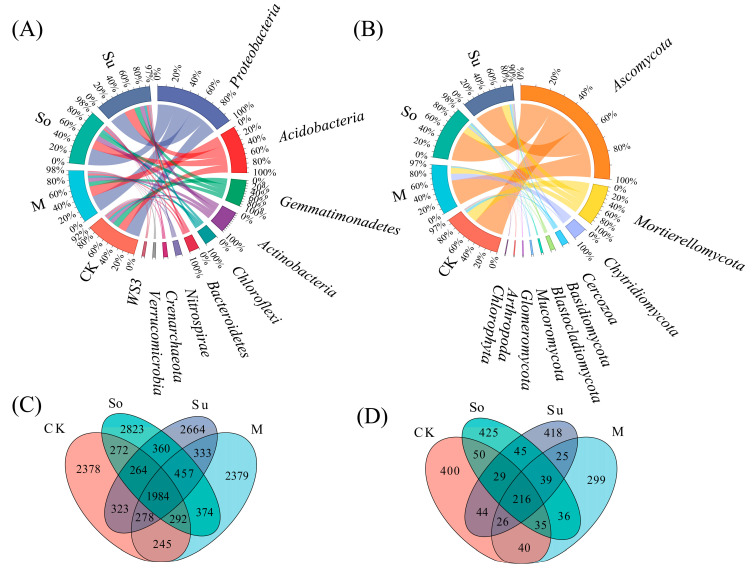
Impact of crop rotation systems on the top 10 bacterial and fungal phylum level community structure in the rhizosphere soil during the seedling stage of *Bupleurum*, and Venn diagrams of bacteria and fungi. Bacterial community (**A**,**C**), fungal community (**B**,**D**).

Under four rotation systems, the top ten dominant phyla in the bacterial community comprised the Proteobacteria, Acidobacteria, Gemmatimonadetes, Actinobacteria, Chloroflexi, Bacteroidetes, Nitrospirae, Crenarchaeota, Verrucomicrobia, and WS3 phyla, collectively accounting for 92% of the total abundance across all phyla, whereas especially So and M were the highest (98%) (Figure 3A). In particular, the top three dominant bacterial community phyla were Proteobacteria (35.33–37.22%), Acidobacteriota (18.78–23.80%), and Gemmatimonadetes (10.25–14.19%). However, the top 10 dominant phyla in the fungal community occupied 90% of the total phylum abundance, comprising the following phyla: Ascomycota, Mortierellomycota, Chytridiomycota, Cercozoa, Basidiomycota, Blastocladiomycota, Mucoromycota, Glomeromycota, Arthropoda, and Chlorophyta, with So being the highest (98%) (Figure 3B). Additionally, the top three dominant fungal community phyla were Ascomycota (60.64–68.60%), Mortierellomycota (15.06–24.33%), and Chytridiomycota (2.64–16.88%).

Collectively, the four rotation patterns resulted in 15,426 bacterial amplicon sequence variants (ASVs) and 2127 fungal ASVs being identified. Of these, soil bacteria accounted for 1984 ASVs, representing 12.86% of the total bacterial ASVs. The total ASVs counts for CK, So, Su, and M were 6036, 6826, 6663, and 6342, respectively. In particular, So had the highest number of unique ASVs, while CK had the lowest (Figure 3C). The total number of soil fungal ASVs was 216, accounting for 10.16% of the total fungal ASVs. The total ASV counts under CK, So, Su, and M were 840, 875, 842, and 716, respectively, with So harboring the highest amount of unique ASVs and M the lowest (Figure 3D).

**Figure 4:**
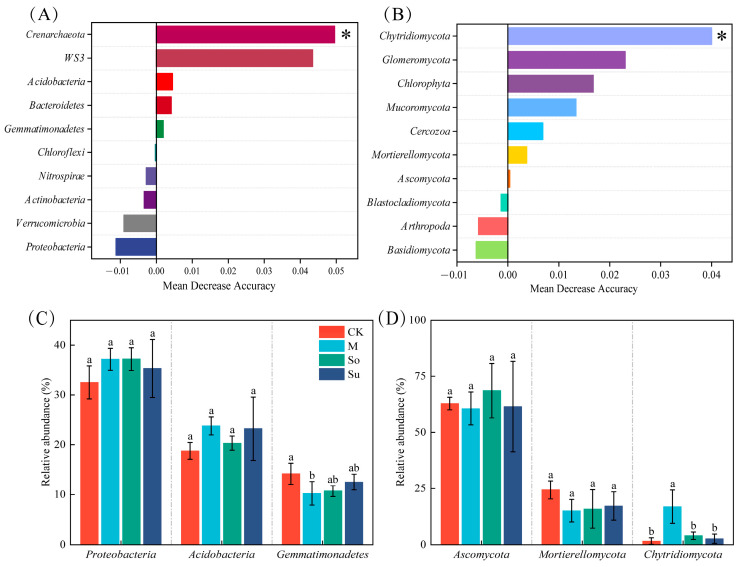
Random forest prediction analysis of the importance and top three of soil bacterial (**A**,**C**) and fungal (**B**,**D**) phylum level community populations under various crop rotation patterns. * means *p* < 0.05. Significant differences among the treatments were indicated by the use of different lowercase letters (*p* < 0.05; Duncan’s test).

The random forest model revealed significantly different bacterial and fungal populations at the phylum level in soil microbial communities across four crop rotation patterns. Among these, the Crenarchaeota exhibited the greatest significance for bacteria, while the Chytridiomycota showed the highest significance for fungi (Figure 4A,B). However, the top three phyla in bacterial and fungal communities that showed significant differences between CK and M were Gemmatimonadetes and Chytridiomycota (Figure 4C,D).

### 3.3. Soil Microbial Alpha and Beta Diversity

**Figure 5:**
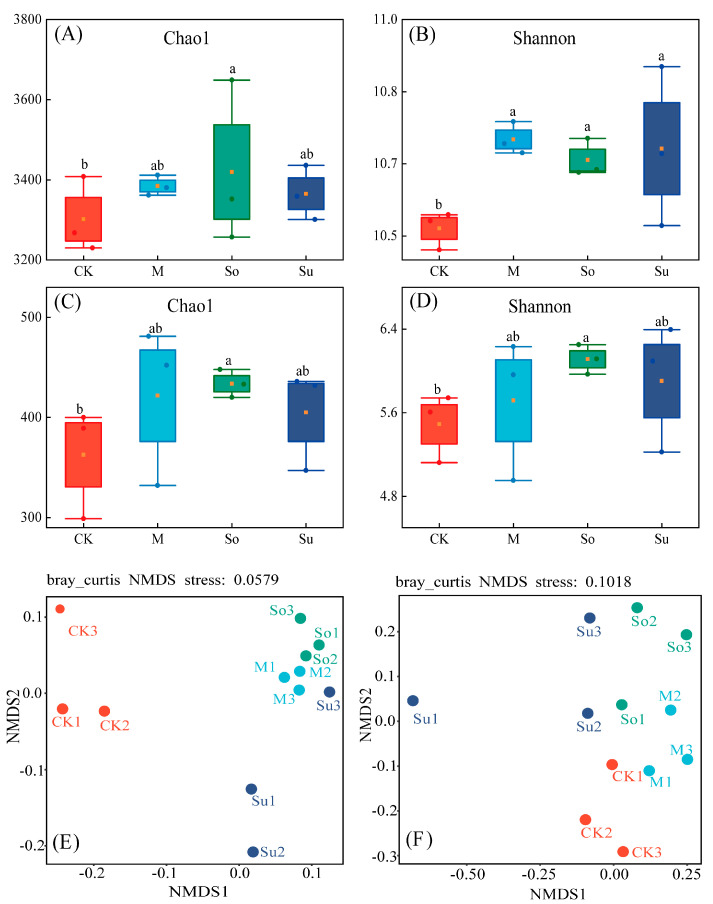
Alpha diversity indices of soil bacterial and fungal community structures under various crop rotation patterns and non-metric multidimensional scaling (NMDS) of bacterial and fungal community using OTU level Bray–Curtis distances represent microbial beta diversity. Bacterial community (**A**,**C**,**E**), fungal community (**B**,**D**,**F**). Different lowercase letters denote variations between treatments. The box represents the interquartile range. The upper and lower whiskers represent the data range. Yellow nodes denote average. Circles of different colors represent sample points.

In comparison with CK, both bacterial and fungal community α diversity (Chao1 index and Shannon index) exhibited a significant increase under So treatment, whereas no remarkable discrepancies in diversity indices were found among So, M, and Su (Figure 5A–D). Based on the analysis of bacterial community similarity, a stress value of 0.0579 (less than 0.1) indicated reliable results. So and M samples exhibited relatively concentrated distributions, indicating high similarity in their community compositions and significant differences from CK (Figure 5E). For fungal community similarity, stress was found to be 0.1018 < 0.2, suggesting that relatively reliable results can be expected. Compared to CK, the So, M, and Su samples clearly showed concentrated distributions and high similarity in their community compositions (Figure 5F).

### 3.4. Co-Occurrences Network Among Microbial Communities

**Figure 6:**
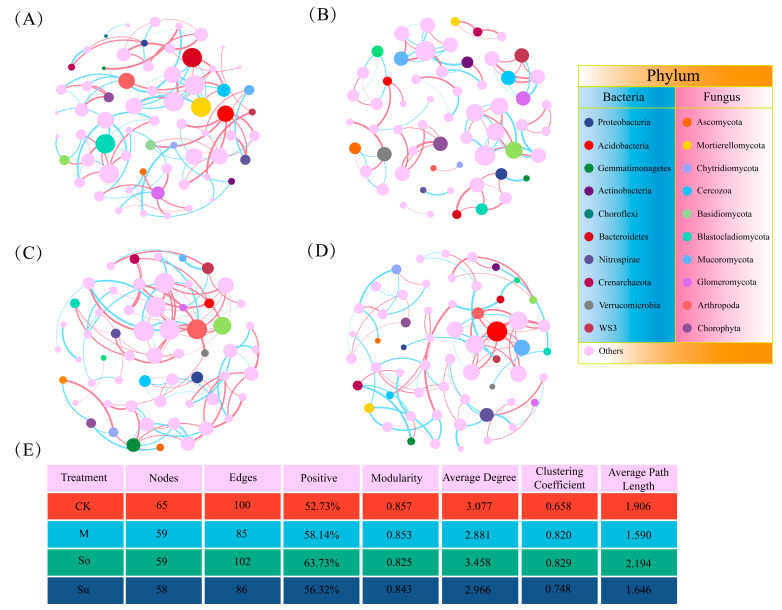
The co-occurrence network relationships between soil bacterial and fungal community under different crop rotation systems. (**A**) CK; (**B**) M; (**C**) So; (**D**) Su. Network nodes denote individual taxonomic units, with the size and color of each node corresponding to its abundance. Network links are represented by pair-wise correlations between nodes, with the thickness being proportional to the r-value. Red and blue links show positive and negative interactions between two nodes, respectively. The microbial coexistence network’s topological features are shown in table (**E**).

To analyze the symbiotic relationships between bacterial and fungal communities, we constructed microbial network diagrams based on four crop rotation patterns, demonstrating distinct topological characteristics (Figure 6). Of these, the soil microbial community symbiosis under the So pattern exhibited the greatest network complexity, as indicated by its edge count, the proportion of positive correlation links, average degree, average clustering coefficient, and average path length. In contrast, the microbial community network under the M treatment had the simplest structure, with the fewest edges and nodes, as well as the lowest average degree and average path length. The CK treatment had the highest number of nodes and modularity, but the fewest positive links. The Su treatment exhibited the lowest number of nodes.

### 3.5. Metabolic Pathways in Microbial Function Prediction

**Figure 7:**
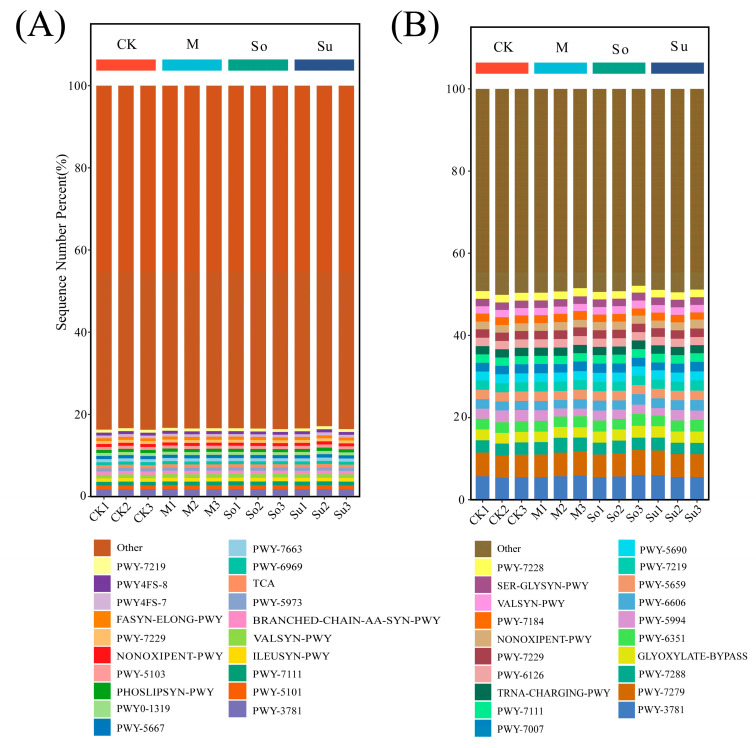
Predicted relative abundance of metabolic pathway functions in soil bacterial (**A**) and fungal (**B**) communities based on MetaCyc across various crop rotation patterns.

The MetaCyc metabolic pathway database was used to conduct an abundance analysis, which was then used to make preliminary functional predictions about soil bacteria and fungi in the rhizosphere of bupleurum root across different rotation patterns. A total of 438 metabolic pathways were annotated across all bacterial communities, and no significant differences were detected in the top 20 pathways overall (Figure 7A; Table 1). Among these, those involved in the amino acid synthesis pathway included BRANCHED-CHAIN-AA-SYN-PWY, ILEUSYN-PWY, PWY-5101, PWY-5103, and VALSYN-PWY. The lipid metabolism pathway involved FASYN-ELONG-PWY, PHOSLIPSYN-PWY, PWY-5667, PWY0-1319, PWY-5973, PWY4FS-7, and PWY4FS-8. The involvement of PWY-7211, PWY-7219, and PWY-7229 in nucleotide synthesis pathways were well documented. The energy regulation pathways included TCA, PWY-6969, PWY-7663, NONOXIPENT-PWY, and PWY-7111.

We annotated all the samples to 73 metabolic pathways in fungal communities, and detected no significant differences across the top 20 pathways overall (Figure 7B; Table 2). The participation of SER-GLYSYN-PWY and VALSYN-PWY is in amino acid synthesis pathways. PWY-5994, PWY-7007, and PWY-7288 are involved in pathways of lipid metabolism. The following compounds were involved in nucleotide synthesis and degradation pathways: PWY-7219, PWY-7229, PWY-6126, PWY-7228, PWY-7184, and PWY-6606. PWY-3781, PWY-7279, PWY-5690, NONOXIPENT-PWY, PWY-7288, and PWY-7111 were involved in pathways that regulated energy. The carbon metabolism pathways were involved by GLYOXYLATE-BYPASS and PWY-5659. Those involved in cofactor and secondary metabolite synthesis pathways included PWY-6351 and PWY-7007. Those involved in protein synthesis pathways included TRNA-CHARGING-PWY.

**Figure 8:**
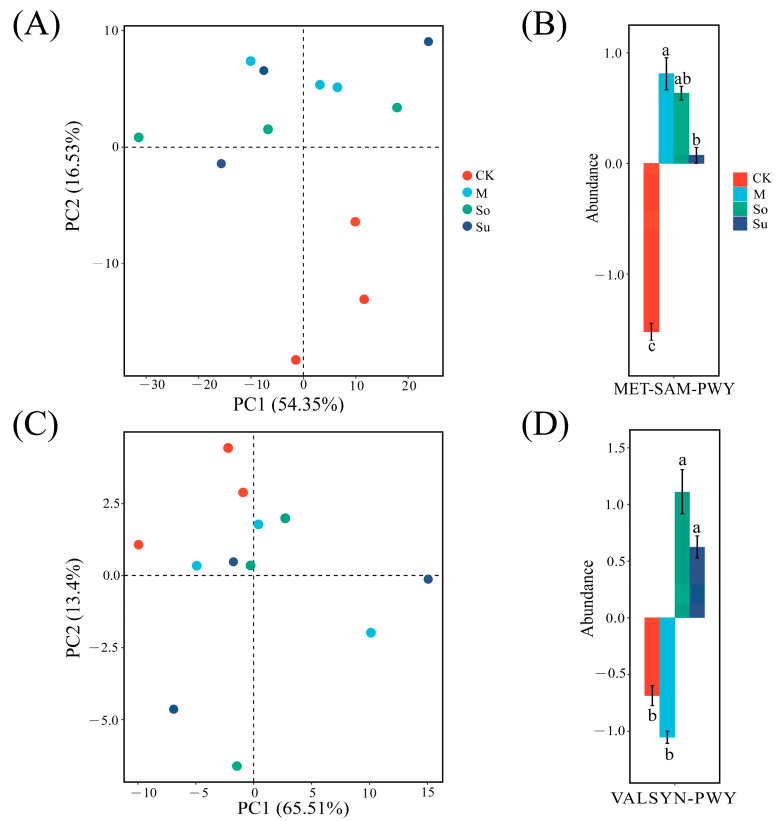
Principal component analysis (PCA) of predicted microbial functional profiles under different crop rotation systems. Points represent individual samples, colored by crop rotation systems. Discriminative metabolic pathways across treatment groups, as verified by Duncan’s test (*p* < 0.05). The vertical axis displays centered pathway abundance values, representing variation from the overall mean. Negative scores signify depletion relative to the mean, whereas positive scores indicate enrichment. Statistical homogenous subsets are denoted by differing lowercase letters. Bacterial community (**A**,**B**), fungal community (**C**,**D**).

Principal component analysis (PCA) was carried out based on the abundance of MetaCyc metabolic pathways in soil bacterial and fungal communities. The results demonstrated that the combination of three preceding crops and the *Bupleurum* rotation pattern performed better than the fallow-*Bupleurum* rotation pattern. Among the predicted functions of bacterial communities, PC1 (54.35%) and PC2 (16.53%) together accounted for 70.88% of the total functional variance. ANOVA and Duncan’s test confirmed that the relative abundance of the methionine synthesis pathway (MET-SAM-PWY) under M and So were greater than average, with no significant difference (Figure 8A,B). In the fungal community functional prediction, PC1 (65.51%) and PC2 (13.4%) collectively accounted for 78.91% of the total functional variation. Following ANOVA and Duncan’s test, the relative abundance values of the valine synthetic pathway (VALSYN-PWY) under So and Su were higher than average, with no significant difference (Figure 8C,D).

### 3.6. Correlation Analysis of Soil Properties, Enzyme Activities, and Soil Microbial Characteristics

**Figure 9:**
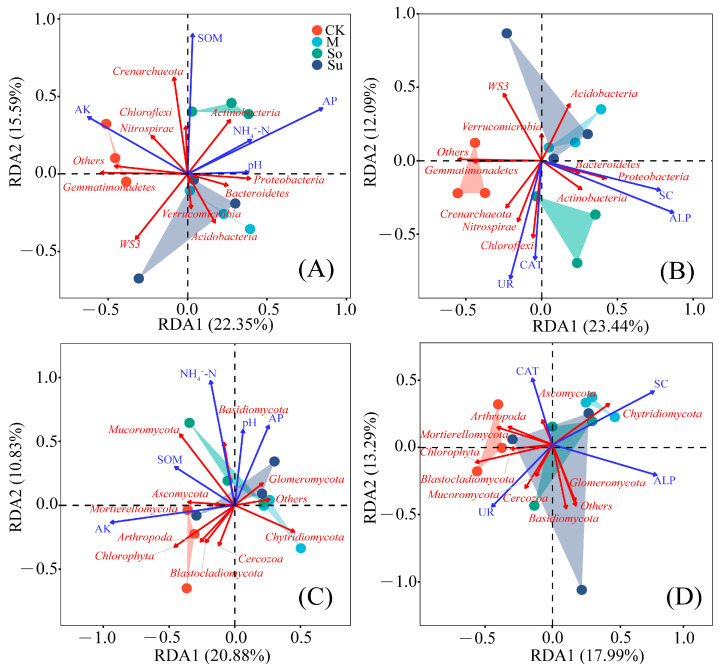
Redundancy analysis (RDA) reflected the relationships among soil bacterial (**A**,**C**) and fungal (**B**,**D**) community phylum level distribution, properties, and enzyme activities.

We analyzed the relationships between soil bacterial communities and soil properties, as well as soil enzyme activities. The two RDA coordinate axes collectively explained 37.94% of the variance in bacterial community composition associated with soil properties (RDA1: 22.35%; RDA2: 15.59%; Figure 9A). Similarly, the two axes together accounted for 35.53% of the variance in enzyme activities (RDA1: 23.44%; RDA2: 12.09%; Figure 9B). SOM, CAT, and UR exhibited positive correlations with Chloroflexi, Actinobacteria, Nitrospirae, and Crenarchaeota, but were adverse correlations with Acidobacteria, Verrucomicrobia, and WS3. In contrast, NH_4_^+^-N, AP, pH, SC, and ALP showed positive associations with Proteobacteria, Actinobacteria, and Bacteroidetes. AK was positively correlated with Chloroflexi, Gemmatimonadetes, Nitrospirae, and Crenarchaeota.

RDA was conducted to determine the relationships of soil fungal communities with soil properties; additionally, it was found that 20.88% of the variation was explained by RDA1 and 10.83% by RDA2, with a total of 31.71% (Figure 9C). Similarly, the analysis of fungal communities in relation to soil enzyme activities showed that the two axes together accounted for 31.28% of the variation (RDA1: 17.99%; RDA2: 13.29%; Figure 9D). NH_4_^+^-N, AP, and pH mainly had positive correlations with Basidiomycota, Mucoromycota, and Glomeromycota. SOM showed positive associations with Ascomycota, Mortierellomycota, Basidiomycota, and Mucoromycota. Positive associations were found among AK and Ascomycota, Mortierellomycota, Arthropoda, Chlorophyta, Cercozoa, and Blastocladiomycota. However, all soil properties were adversely associated with Chytridiomycota. SC was positively associated with Chytridiomycota. ALP was positively associated with Chytridiomycota, Basidiomycota, and Glomeromycota. CAT showed positive relationships with Ascomycota, Mortierellomycota, and Arthropoda. UR was positively associated with Blastocladiomycota, Chlorophyta, Mucoromycota, and Cercozoa.

**Figure 10:**
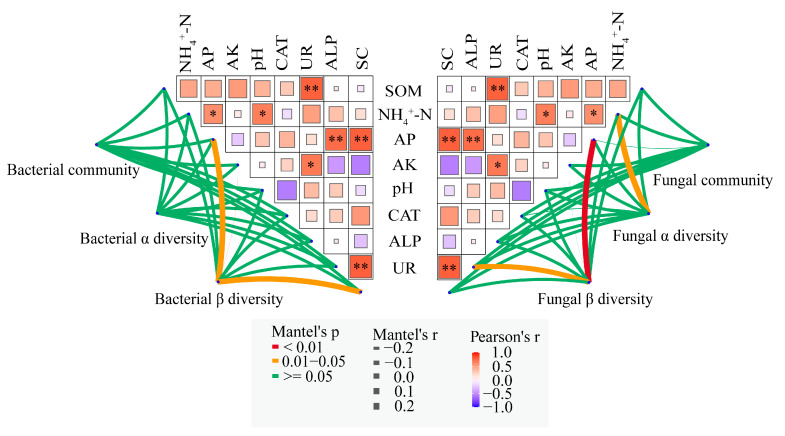
Analyzing the relationships of soil properties, enzyme activities and soil bacterial community composition, α and β diversity based on Mantel test. * means *p* < 0.05 and ** means *p* < 0.01.

The Pearson’s correlation coefficients demonstrated that there were significant positive correlations among pH with NH_4_^+^-N, UR with SOM, AK, SC, and AP with NH_4_^+^-N, ALP, and SC. Mantel tests revealed that soil bacterial community β diversity was primarily significantly interrelated with AP content and SC activity. Soil fungal community α diversity showed a significant positive association with NH_4_^+^-N content. Furthermore, soil fungal community β diversity was mainly influenced by AP content and UR activity (Figure 10).

## 4. Discussion

### 4.1. Effects of Different Crop Rotation Patterns on Soil Properties and Enzyme Activities in the Rhizosphere of Bupleurum Seedlings

Crop rotation is considered to be a productive cultivation method for alleviating continuous cropping obstacles, as residual effects are exerted by preceding crops on succeeding crops, thereby promoting their growth [29]. This study indicated that So dramatically raised the content of SOM (11.24%), NH_4_^+^-N (27.22%), AP (75.22%), and pH value (2.18%), compared to CK. These results were in agreement with those of Oliveira et al. [30], who attributed this to the differential impacts of crop root exudates, plant root residues, and litter on pathogens [31]. Compared with M and Su, So significantly increased the content of SOM, NH_4_^+^-N, AP, and AK. This was primarily due to different preceding crops altering the input and chemical composition of plant residues in the soil, which significantly influenced soil properties [32]. Compared to maize and sunflowers, which are grass and oilseed crops, respectively, soybeans, as a legume, possessed unique biological nitrogen-fixing capabilities. Firstly, soybeans formed a symbiotic relationship with nitrogen-fixing bacteria. Secondly, their root systems released organic acids that encouraged the conversion and uptake of soil nitrogen, thus raising the total amount of nitrogen in the soil [33] and affecting phosphorus movement and conversion [34]. Both Dou et al. [31] and Li et al. [35] demonstrated that maize cultivation had no significant impact on soil nitrogen content. Appropriate crop rotation improved soil physicochemical properties [36] and enhanced soil enzyme activities [37]. Our research showed that, in comparison to CK, M and So significantly elevated SC and ALP activities. So significantly increased UR activity compared to M (Figure 2). These findings were aligned with those of Fan et al. [38], who indicated that leguminous crops had a greater stimulatory effect on soil enzyme activity than grass crops within a rotational system. This may be because legumes promoted soil biochemical processes, thereby enhancing soil enzyme activity. Consequently, So enhanced soil properties and enzyme activities, and then optimized the microenvironment.

### 4.2. Effects of Different Crop Rotation Patterns on the Composition and Diversity of Soil Microbial Communities in the Rhizosphere of Bupleurum Seedlings

Different preceding crops typically resulted in significant differences in soil microbial community composition and diversity [39]. Our research indicated that top 10 dominant phyla in the bacterial community collectively accounted for 92% of the total abundance across all phyla, and especially So and M were the highest (98%) (Figure 3A). In particular, the top three dominant bacterial community phyla were Proteobacteria (35.33–37.22%), Acidobacteriota (18.78–23.80%), and Gemmatimonadetes (10.25–14.19%). However, the top 10 dominant phyla in the fungal community occupied 90% of the total phylum abundance, with So being the highest (98%) (Figure 3B). Additionally, top three dominant fungal community phyla were Ascomycota (60.64–68.60%), Mortierellomycota (15.06–24.33%), and Chytridiomycota (2.64–16.88%). This finding was corroborated by Yan et al. [40] and Dou et al. [31] who suggested that root exudates from different crops can drive significant differences in rhizosphere microbial communities and selectively regulate their composition [41,42]. The total ASVs counts for bacterial and fungal communities under So were 6826 and 875, respectively, with So exhibiting the highest number of unique ASVs and CK the lowest (Figure 3C,D). This further indicated soybean’s greater soil-improving potential as a preceding crop [43], likely attributable to its symbiotic nitrogen-fixing capacity and the positive impact of organic inputs on microbial communities [44]. However, the relative abundance of Gemmatimonadetes and Chytridiomycota among the top three bacterial and fungal phyla varied across four rotation patterns. This discrepancy likely stemmed from the fact that different preceding crops shaped specific microenvironments through root exudates, thereby influencing the abundance of associated microbial groups. This study indicates that, compared to other treatments, So enhances the α diversity of bacterial and fungal communities (chao1 and Shannon indices), and that So and M exhibited high similarity in microbial community composition. These outcomes were in concordance with the results of Oberholster et al. [45] and Geng et al. [46]. The mechanism involved soybean roots secreting flavonoid compounds that recruited specific microorganisms [29], thereby enhancing soil microbial diversity in the rhizosphere. Therefore, So optimized the soil microenvironment, improving both bacterial and fungal community structure and diversity holistically.

### 4.3. Effects of Different Crop Rotation Patterns on Networks’ Symbiotic Relationships and Functional Prediction of Soil Microbial Community in the Rhizosphere of Bupleurum Seedlings

In general, soil microbial communities with higher alpha diversity contributed to greater ecosystem stability [47]. Wang et al. [48] indicated that the complexity of microbial networks increased with greater diversity of key species. Our study showed that the rhizosphere microbial symbiotic network under So was highly complex. This differentiated species co-occurrence pattern likely stemmed from variations in the quantity and quality of rhizosphere sediments and crop residues from different preceding crops (maize, soybean, and sunflower). These variations altered soil physicochemical properties, thereby influencing microbial niches and ultimately regulating the intricacies of bacterial and fungal community interaction networks [49,50,51]. Strengthening the multifunctionality and stability of soil microbial communities involved enhancing microbial diversity and interspecies network complexity [52,53]. No clear discrepancies were found in the predicted functional abundance of the top twenty metabolic pathways in bacterial and fungal communities across the four rotation patterns. PCA results indicated that the So, M, and Su performed better overall than the CK, with particularly prominent metabolic pathways observed in the bacterial communities under So (MET-SAM-PWY: methionine synthesis pathway) and in the fungal communities (VALSYN-PWY: valine synthesis pathway). This phenomenon may be due to ecological redundancy in soil microbial functions, whereby localized changes in diversity did not significantly impact the overall biochemistry of the system [54,55]. In particular, amino acid metabolism reflected the overall metabolic activity of microorganisms and was closely linked to soil nutrient accumulation and quality improvement [56]. This study also confirmed that So improved soil properties and increased enzyme activities. Consequently, So enhanced the stability and multifunctionality of the bupleurum farmland ecosystem by establishing intricate symbiotic networks among bacterial and fungal communities and fortifying the functional capacity of amino acid synthetic metabolic pathways.

### 4.4. Correlation Analysis Between Soil Microorganisms, Soil Properties, and Enzyme Activities

Soil microorganisms significantly influence soil physicochemical properties and enzyme activities [57]. Our study revealed there were significant positive correlations among pH with NH_4_^+^-N, UR with SOM, AK, SC, and AP with NH_4_^+^-N, ALP, and SC. RDA analysis revealed that the primary drivers of bacterial community composition were soil properties (AP and pH) and enzyme activities (SC and ALP), while the prominent drivers of fungal community composition were soil properties (SOM and AK) and enzyme activities (SC and CAT). Furthermore, soil property AP significantly influenced both bacterial and fungal community β diversity. These findings were supported by previous studies [25,29,58,59,60,61]. The structure and function of microorganisms were typically dominated by key taxa [62]. As such, the phyla Myxobacteria, Bacteroidetes, and Basidiomycota played crucial roles in amino acid metabolism and carbon metabolism [63]. Acidobacteria and Bacteroidetes were involved in the metabolism of carbon sources and inorganic and organic nitrogen sources [64,65]. Mortierellomycota and Chytridiomycota facilitated the decomposition of organic matter and detritus, enhanced the availability of soil nutrients, participated in the carbon and nitrogen cycles of the soil, and improved crop resilience [66,67].

## 5. Conclusions

Our results demonstrated that, compared to CK, So significantly improved soil properties and enzyme activities. So not only optimized the structure of soil bacterial and fungal communities, but also markedly enhanced microbial α diversity. The bacterial and fungal community compositions of So and M were found to be highly similar, displaying intricate symbiotic relationships within the soil microbial community network. No clear discrepancies were detected in the predicted functional abundance of the top twenty metabolic pathways for bacterial and fungal communities across four rotation patterns. However, So, M, and Su rotations outperformed CK. Bacterial metabolic pathways MET-SAM-PWY and fungal metabolic pathways VALSYN-PWY were particularly prominent under So. RDA analysis indicated that the primary drivers of bacterial community composition were soil properties (available phosphorus, AP, and pH) and enzyme activities (sucrase, SC, and alkaline phosphatase activities), and the primary drivers of fungal community composition were soil properties (soil organic matter and available potassium) and enzyme activities (SC and catalase activities). Mantel analysis revealed that AP significantly impacted both bacterial and fungal community β diversity. In summary, the soybean-*Bupleurum* rotation is the optimal cultivation method for *Bupleurum* and has significant future development potential for the *Bupleurum* industry.

## Figures and Tables

**Table 1:** Annotation of the top 20 metabolic pathway abundances in soil bacterial communities at the rhizosphere of *Bupleurum* under various crop rotation patterns.

Pathway	Description
BRANCHED-CHAIN-AA-SYN-PWY	superpathway of branched amino acid biosynthesis
FASYN-ELONG-PWY	fatty acid elongation saturated
ILEUSYN-PWY	L-isoleucine biosynthesis I (from threonine)
NONOXIPENT-PWY	pentose phosphate pathway (non-oxidative branch)
PHOSLIPSYN-PWY	superpathway of phospholipid biosynthesis I (bacteria)
PWY-5101	L-isoleucine biosynthesis II
PWY-5103	L-isoleucine biosynthesis III
PWY-5667	CDP-diacylglycerol biosynthesis I
PWY-5973	cis-vaccenate biosynthesis
PWY-6969	TCA cycle V (2-oxoglutarate:ferredoxin oxidoreductase)
PWY-7111	pyruvate fermentation to isobutanol (engineered)
PWY-7211	superpathway of pyrimidine deoxyribonucleotides de novo biosynthesis
PWY-7219	adenosine ribonucleotides de novo biosynthesis
PWY-7229	superpathway of adenosine nucleotides de novo biosynthesis I
PWY-7663	gondoate biosynthesis (anaerobic)
PWY0-1319	CDP-diacylglycerol biosynthesis II
PWY4FS-7	phosphatidylglycerol biosynthesis I (plastidic)
PWY4FS-8	phosphatidylglycerol biosynthesis II (non-plastidic)
TCA	TCA cycle I (prokaryotic)
VALSYN-PWY	L-valine biosynthesis

**Table 2:** Annotation of the top 20 metabolic pathway abundances in soil fungal communities at the rhizosphere of *Bupleurum* under various crop rotation patterns.

Pathway	Description
GLYOXYLATE-BYPASS	glyoxylate cycle
NONOXIPENT-PWY	pentose phosphate pathway (non-oxidative branch)
PWY-3781	aerobic respiration I (cytochrome c)
PWY-5659	GDP-mannose biosynthesis
PWY-5690	TCA cycle II (plants and fungi)
PWY-5994	palmitate biosynthesis I (animals and fungi)
PWY-6126	superpathway of adenosine nucleotides de novo biosynthesisII
PWY-6351	D-myo-inositol (1,4,5)-trisphosphate biosynthesis
PWY-6606	guanosine nucleotides degradation II
PWY-7007	methyl ketone biosynthesis
PWY-7111	pyruvate fermentation to isobutanol (engineered)
PWY-7184	pyrimidine deoxyribonucleotides de novo biosynthesis I
PWY-7219	adenosine ribonucleotides de novo biosynthesis
PWY-7228	superpathway of guanosine nucleotides de novo biosynthesis I
PWY-7229	superpathway of adenosine nucleotides de novo biosynthesis I
PWY-7279	aerobic respiration II (cytochrome c) (yeast)
PWY-7288	fatty acid &beta;-oxidation (peroxisome, yeast)
SER-GLYSYN-PWY	superpathway of L-serine and glycine biosynthesis I
TRNA-CHARGING-PWY	tRNA charging
VALSYN-PWY	L-valine biosynthesis

## Data Availability

The original contributions presented in this study are included in the article. Further inquiries can be directed to the corresponding authors.

## References

[1] Yang F., Dong X., Yin X., Wang W., You L., Ni J. (2017). *Radix Bupleuri*: A review of traditional uses, botany, phytochemistry, pharmacology, and toxicology. Biomed. Res. Int..

[2] Wang L., Chen M., Lam P.-Y., Dini-Andreote F., Dai L., Wei Z. (2022). Multifaceted roles of flavonoids mediating plant-microbe interactions. Microbiome.

[3] Sun J., Li X., Qu Z., Wang H., Cheng Y., Dong S., Zhao H. (2023). Comparative proteomic analysis reveals novel insights into the continuous cropping induced response in *Scrophularia ningpoensis*. J. Sci. Food Agric..

[4] Yu T., Hou X., Fang X., Razavi B., Zang H., Zeng Z., Yang Y. (2024). Short-term continuous monocropping reduces peanut yield mainly via altering soil enzyme activity and fungal community. Environ. Res..

[5] Liu S., Wang Z., Niu J., Dang K., Zhang S., Wang S., Wang Z. (2021). Changes in physicochemical properties, enzymatic activities, and the microbial community of soil significantly influence the continuous cropping of *Panax quinquefolius* L. (American ginseng). Plant Soil.

[6] He S., Lv M., Wang R., Li N., Wang T., Shi W., Gao Z., Li X. (2024). Long-term garlic—maize rotation maintains the stable garlic rhizosphere microecology. Environ. Microbiome.

[7] Zhao W., Liu R., Wang Z., Feng Y., Xue K., Liu K., Xue Z., Cao W., Fu L., Yin M. (2024). Effects of rotation with a green manure crop on soil quality and microbial nutrient limitation in a tobacco field in Yunnan. Acta Pratacult. Sin..

[8] Li B., Zhang Q., Chen Y., Su Y., Sun S., Chen G. (2021). Different crop rotation systems change the rhizosphere bacterial community structure of *Astragalus membranaceus* (Fisch) Bge. var. *mongholicus* (Bge.) Hsiao. Appl. Soil Ecol..

[9] Qin X., Jiang J., Wei Q., Zhang X., Zhou C., Zhao J., Huang L., Guo S., Luo S., Ding J. (2024). Effects of rotation and apply bioorganic fertilizer on soil microbial characteristics of pineapple under continuous cropping. Soil Fertil. Sci. China.

[10] Li X., Zhang Y., Tian Z., Wu G. (2017). Difference analysis of soil nutrients, enzymatic activities and microbial community structure between eggplant continuous cropping and rotation. J. Zhejiang Univ. Agric. Life Sci..

[11] Wang F., Zhang X., Wei M., Wang Y., Liang Z., Xia P. (2022). Appropriate crop rotation alleviates continuous cropping barriers by changing rhizosphere microorganisms in *Panax notoginseng*. Rhizosphere.

[12] Lu J., Liu Y., Zou X., Zhang X., Yu X., Wang Y., Si T. (2024). Rotational strip peanut/cotton intercropping improves agricultural production through modulating plant growth, root exudates, and soil microbial communities. Agric. Ecosyst. Environ..

[13] Wang X., Chi Y., Song S. (2024). Important soil microbiota’s effects on plants and soils: A comprehensive 30-year systematic literature review. Front. Microbiol..

[14] Zhou Y., Yang Z., Liu J., Li X., Wang X., Dai C., Zhang T., Carrión V.J., Wei Z., Cao F. (2023). Crop rotation and native microbiome inoculation restore soil capacity to suppress a root disease. Nat. Commun..

[15] Liu L., Ma L., Zhu M., Liu B., Liu X., Shi Y. (2023). Rhizosphere microbial community assembly and association networks strongly differ based on vegetation type at a local environment scale. Front. Microbiol..

[16] Hu Q., Liu Y., Wang W., Wang Q., Wang H., Chu F. (2021). Effects of Sweet Potato Rotation and Intercropping on the Microbial Community of Rhizosphere Soil. Crops.

[17] Wang L., Liu S., Tian G., Pan Y., Wang H., Qiu G., Li F., Pang Z., Ding K., Zhang J. (2025). Impacts of continuous potato cropping on soil microbial assembly processes and spread of potato common scab. Appl. Soil Ecol..

[18] Zhang X., Duan H., Wang M., Li H., Lu A., Wang Y. (2018). Effects of rotation and continuous cropping on soil microflora and diversity in tobacco field. Soil Fertil. Sci. China.

[19] Liu H., Su Y., Fang L., Luo L., Wang L., Zhang Z., Zhu S., Yang M. (2021). The effect and mechanism of fennel crop rotation on soil bacterial community to alleviate replant failure of *Panax notoginseng*. Chin. J. Biol. Control.

[20] Wang Y., Ji H., Wang R., Guo S., Gao C. (2017). Impact of root diversity upon coupling between soil C and N accumulation and bacterial community dynamics and activity: Result of a 30 year rotation experiment. Geoderma.

[21] Kalembasa S.J., Jenkinson D.S. (1973). A comparative study of titrimetric and gravimetric methods for the determination of organic carbon in soil. J. Sci. Food Agric..

[22] Lu R. (2000). Analysis Methods of Soil Agricultural Chemistry.

[23] Jones J. (2018). Soil Analysis Handbook of Reference Methods.

[24] Olsen S.R. (1954). Estimation of Available Phosphorus in Soils by Extraction with Sodium Bicarbonate.

[25] Guo C., Yang C., Fu J., Song Y., Chen S., Li H., Ma C. (2024). Effects of crop rotation on sugar beet growth through improving soil physicochemical properties and microbiome. Ind. Crops Prod..

[26] Li Y., Chen J., Dong Q., Feng H., Siddique K.H. (2022). Plastic mulching significantly improves soil enzyme and microbial activities without mitigating gaseous N emissions in winter wheat-summer maize rotations. Field Crops Res..

[27] Callahan B.J., McMurdie P.J., Rosen M.J., Han A.W., Johnson A.J.A., Holmes S.P. (2016). DADA2: High-resolution sample inference from Illumina amplicon data. Nat. Methods.

[28] Langille M.G., Zaneveld J., Caporaso J.G., McDonald D., Knights D., Reyes J.A., Clemente J.C., Burkepile D.E., Vega Thurber R.L., Knight R. (2013). Predictive functional profiling of microbial communities using 16S rRNA marker gene sequences. Nat. Biotechnol..

[29] Wang Y., Zhang H., Zhang Y., Fei J., Xiangmin R., Peng J., Luo G. (2023). Crop rotation-driven changes in rhizosphere metabolite profiles regulate soil microbial diversity and functional capacity. Agric. Ecosyst. Environ..

[30] Lago-Olveira S., Ouhemi H., Idrissi O., Moreira M.T., González-García S. (2024). Promoting more sustainable agriculture in the Moroccan drylands by shifting from conventional wheat monoculture to a rotation with chickpea and lentils. Clean. Environ. Syst..

[31] Dou Y., Yu S., Liu S., Cui T., Huang R., Wang Y., Wang J., Tan K., Li X. (2025). Crop rotations reduce pathogenic fungi compared to continuous cropping. Rhizosphere.

[32] Pituello C., Polese R., Morari F., Berti A. (2016). Outcomes from a long-term study on crop residue effects on plant yield and nitrogen use efficiency in contrasting soils. Eur. J. Agron..

[33] Wang H., Chen J., Wang Y., Guo J., Shao R., Wang S., Yang Q. (2025). Metagenomic study on microbial function in increasing total nitrogen content in soil under crop rotation systems. Appl. Soil Ecol..

[34] Kalembasa D., Szukała J., Symanowicz B., Kalembasa S., Faligowska A., Becher M. (2021). Amount of biologically nitrogen fixed by faba bean and its uptake by winter wheat determined by 15N ID method. Arch. Agron. Soil Sci..

[35] Li R., He Z., He P., Yang R., Ma D., Li Y., Xiang Y., Sun W., Zhu X., Zhang Z. (2025). Crop diversification improves productivity and soil health by regulating fungal diversity. Soil Tillage Res..

[36] Wang Y., Shi M., Zhang R., Zhang W., Liu Y., Sun D., Wang X., Qin S., Kang Y. (2023). Legume-potato rotation affects soil physicochemical properties, enzyme activity, and rhizosphere metabolism in continuous potato cropping. Chem. Biol. Technol. Agric..

[37] González-Chávez M.d.C.A., Aitkenhead-Peterson J.A., Gentry T.J., Zuberer D., Hons F., Loeppert R. (2010). Soil microbial community, C, N, and P responses to long-term tillage and crop rotation. Soil Tillage Res..

[38] Fan J., Hao M. (2003). Study on long-term experiment of crop rotation and fertilizationin the Loess PlateauI I. Effect of crop rotation and continuous planting on soil enzyme activities. J. Plant Nutr. Fertil..

[39] Zhang H., Luo G., Wang Y., Fei J., Xiangmin R., Peng J., Tian C., Zhang Y. (2023). Crop rotation-driven change in physicochemical properties regulates microbial diversity, dominant components, and community complexity in paddy soils. Agric. Ecosyst. Environ..

[40] Yan H., Zhu L., Wang Y., Zhang S., Liu P., Dong T.T., Wu Q., Duan J.-A. (2021). Comparative metagenomics analysis of the rhizosphere microbiota influence on Radix Angelica sinensis in different growth soil environments in China. Food Sci. Technol..

[41] Yuan Y., Zuo J., Zhang H., Zu M., Liu S. (2022). The Chinese medicinal plants rhizosphere: Metabolites, microorganisms, and interaction. Rhizosphere.

[42] Gong X., Feng Y., Dang K., Jiang Y., Qi H., Feng B. (2023). Linkages of microbial community structure and root exudates: Evidence from microbial nitrogen limitation in soils of crop families. Sci. Total Environ..

[43] Zhao J., Yang Y., Zhang K., Jeong J., Zeng Z., Zang H. (2020). Does crop rotation yield more in China? A meta-analysis. Field Crops Res..

[44] Lyu H., Yu A., Chai Q., Wang F., Wang Y., Wang P., Shang Y., Yang X. (2025). Enhancing soil quality and crop yield by increasing dominant bacterial abundance and reducing bacterial diversity under no-tillage with total green manure incorporation. Agric. Ecosyst. Environ..

[45] Oberholster T., Vikram S., Cowan D., Valverde A. (2018). Key microbial taxa in the rhizosphere of sorghum and sunflower grown in crop rotation. Sci. Total Environ..

[46] Geng S., Tan J., Li L., Miao Y., Wang Y. (2023). Legumes can increase the yield of subsequent wheat with or without grain harvesting compared to Gramineae crops: A meta-analysis. Eur. J. Agron..

[47] Shu X., He J., Zhou Z., Xia L., Hu Y., Zhang Y., Zhang Y., Luo Y., Chu H., Liu W. (2022). Organic amendments enhance soil microbial diversity, microbial functionality and crop yields: A meta-analysis. Sci. Total Environ..

[48] Wang X., Teng Y., Wang X., Li X., Luo Y. (2022). Microbial diversity drives pyrene dissipation in soil. Sci. Total Environ..

[49] Xie Z., Yu Z., Li Y., Wang G., Liu X., Tang C., Lian T., Adams J., Liu J., Liu J. (2022). Soil microbial metabolism on carbon and nitrogen transformation links the crop-residue contribution to soil organic carbon. Npj Biofilms Microbiomes.

[50] Nannipieri P., Hannula S.E., Pietramellara G., Schloter M., Sizmur T., Pathan S.I. (2023). Legacy effects of rhizodeposits on soil microbiomes: A perspective. Soil Biol. Biochem..

[51] Nwachukwu B.C., Ayangbenro A.S., Babalola O.O. (2023). Structural diversity of bacterial communities in two divergent sunflower rhizosphere soils. Ann. Microbiol..

[52] Martiny A.C., Treseder K., Pusch G. (2013). Phylogenetic conservatism of functional traits in microorganisms. ISME J..

[53] Konopka A., Lindemann S., Fredrickson J. (2015). Dynamics in microbial communities: Unraveling mechanisms to identify principles. ISME J..

[54] Nannipieri P., Ascher J., Ceccherini M., Landi L., Pietramellara G., Renella G. (2003). Microbial diversity and soil functions. Eur. J. Soil Sci..

[55] Yi M., Zhang L., Li Y., Qian Y. (2022). Structural, metabolic, and functional characteristics of soil microbial communities in response to benzo [a] pyrene stress. J. Hazard. Mater..

[56] Geisseler D., Horwath W.R., Joergensen R.G., Ludwig B. (2010). Pathways of nitrogen utilization by soil microorganisms–A review. Soil Biol. Biochem..

[57] Zou X., Liu Y., Huang M., Li F., Si T., Wang Y., Yu X., Zhang X., Wang H., Shi P. (2023). Rotational strip intercropping of maize and peanut enhances productivity by improving crop photosynthetic production and optimizing soil nutrients and bacterial communities. Field Crops Res..

[58] Ouyang Y., Reeve J.R., Norton J.M. (2022). The quality of organic amendments affects soil microbiome and nitrogen-cycling bacteria in an organic farming system. Front. Soil Sci..

[59] Kumar M., Ansari W.A., Zeyad M.T., Singh A., Chakdar H., Kumar A., Farooqi M.S., Sharma A., Srivastava S., Srivastava A.K. (2023). Core microbiota of wheat rhizosphere under Upper Indo-Gangetic plains and their response to soil physicochemical properties. Front. Plant Sci..

[60] Yuan L., Gao Y., Mei Y., Liu J., Kalkhajeh Y.K., Hu H., Huang J. (2023). Effects of continuous straw returning on bacterial community structure and enzyme activities in rape-rice soil aggregates. Sci. Rep..

[61] Yang H., Cheng L., Che L., Su Y., Li Y. (2024). Nutrients addition decreases soil fungal diversity and alters fungal guilds and co-occurrence networks in a semi-arid grassland in northern China. Sci. Total Environ..

[62] Banerjee S., Schlaeppi K., Van Der Heijden M.G. (2018). Keystone taxa as drivers of microbiome structure and functioning. Nat. Rev. Microbiol..

[63] Tan Y., Wang J., He Y., Yu X., Chen S., Penttinen P., Liu S., Yang Y., Zhao K., Zou L. (2023). Organic fertilizers shape soil microbial communities and increase soil amino acid metabolites content in a blueberry orchard. Microb. Ecol..

[64] Liu B., Cheng X., He X., Bei Q., Dai Y., Wang Y., Zhu B., Zhang K., Tian X., Duan M. (2022). Effects of bio-mulching on wheat soil microbial community and carbon utilization efficiency in southwest China. Catena.

[65] Araujo A.S.F., de Pereira A.P.d.A., Antunes J.E.L., Oliveira L.M.d.S., de Melo W.J., Rocha S.M.B., do Amorim M.R., Araujo F.F., Melo V.M.M., Mendes L.W. (2021). Dynamics of bacterial and archaeal communities along the composting of tannery sludge. Environ. Sci. Pollut. Res..

[66] Ji L., Nasir F., Tian L., Chang J., Sun Y., Zhang J., Li X., Tian C. (2021). Outbreaks of root rot disease in different aged American ginseng plants are associated with field microbial dynamics. Front. Microbiol..

[67] Tarin M.W.K., Fan L., Xie D., Tayyab M., Rong J., Chen L., Muneer M.A., Zheng Y. (2021). Response of soil fungal diversity and community composition to varying levels of bamboo biochar in red soils. Microorganisms.

